# Effects of headgear timing on dental arch changes from 7 to 18 years of age: a follow-up study

**DOI:** 10.1093/ejo/cjad045

**Published:** 2023-08-22

**Authors:** Matti Hannula, Mimmi Tolvanen, Pertti Pirttiniemi, Kirsi Pirilä-Parkkinen, Johanna Julku

**Affiliations:** Research Unit of Population Health, Faculty of Medicine, University of Oulu, PO Box 5000, FIN-90014 Oulu, Finland; Medical Research Center, Oulu University Hospital, University of Oulu, PO Box 8000, FI-90014 Oulu, Finland; Faculty of Medicine, University of Oulu, PO Box 8000, FI-90014 Oulu, Finland; Research Unit of Population Health, Faculty of Medicine, University of Oulu, PO Box 5000, FIN-90014 Oulu, Finland; Medical Research Center, Oulu University Hospital, University of Oulu, PO Box 8000, FI-90014 Oulu, Finland; Oral and Maxillofacial Department, Oulu University Hospital, PO Box 10, FI-90029 OYS, Oulu, Finland; Medical Research Center, Oulu University Hospital, University of Oulu, PO Box 8000, FI-90014 Oulu, Finland; Oral and Maxillofacial Department, Oulu University Hospital, PO Box 10, FI-90029 OYS, Oulu, Finland; Medical Research Center, Oulu University Hospital, University of Oulu, PO Box 8000, FI-90014 Oulu, Finland; Oral and Maxillofacial Department, Oulu University Hospital, PO Box 10, FI-90029 OYS, Oulu, Finland

**Keywords:** Headgear, timing, dental arch, long-term

## Abstract

**Background:**

Dental arch effects after cervical headgear (CHG) treatment have been researched from several different perspectives. However, the long-term effects of CHG timing are still unknown.

**Objectives:**

To analyse the long-term effects of CHG timing on dental arches.

**Material and methods:**

A total of 67 children with Angle Class II malocclusion comprised the study group in this trial. The participants were randomized into two equal-sized groups. In the early group (EG, *n* = 33), treatment was started after the eruption of the first upper molars. In the later-timed group (LG, *n* = 34), treatment was started 18 months later compared with the early group. Long-term effects were measured from dental casts taken at five time points between 7 and 18 years of age.

**Results:**

The total maxillary dental arch length was achieved earlier, the gained length persisted better in the long term, and significantly more space was achieved in EG compared with LG (*P* = .048). The intermolar width in the maxillary dental arch was more stable and was reached earlier in EG compared with LG (*P* = .002). The results showed that in terms of total mandibular arch length increases, EG males benefited the most and LG females the least from CHG treatment (*P* = .031).

**Conclusions:**

Both genders benefited from earlier CHG treatment. The maxillary dental arches remained longer, and the final width was gained earlier in EG compared with LG.

## Introduction

The occlusion and dental arches change dynamically throughout the human lifespan. Dental arches develop and expand from childhood to adolescence, along with craniofacial growth, dentoalveolar growth, and eruption of primary and permanent teeth. Transversal growth of the dental arches is finished before 15 years of age [1]. Maxillary dental arch length increases until 10 years of age and mandibular dental arch until 7 years of age in girls and 10 years of age in boys [2]. Later, after adolescence, dental arch dimensions decrease slightly as part of natural dentoalveolar changes in normal occlusion [2]. Dental arch width is known to be more stable after growth than dental arch length [2].

Angle Class II malocclusion is one of the most prevalent malocclusions worldwide after Class I malocclusion and dental crowding [3]. Narrow dental arches are often related to Class II malocclusion, especially in early mixed dentition, and spontaneous correction during growth cannot be expected [4]. Dental crowding is also associated with relatively narrow and short dental arches [5].

The aim of orthodontic treatment is to normalize occlusion and enable normal dentofacial growth and development. Cervical headgear (CHG) is a well-known and reported orthodontic appliance to correct Class II malocclusion and dental crowding [6–8]. CHG distalizes the upper first molars [9–12] and extends the length of the maxillary dental arch [13–16]. Distal movement of upper canines can also be obtained as a result of CHG treatment [10]. The expanding CHG increases intermolar and intercanine widths in the upper dental arch [13–16]. According to some studies, the mandibular dental arch widens spontaneously with CHG treatment [13–15, 17] and a length extension can be achieved [13, 16]. It has also been suggested that CHG works synergistically with growth to produce positive changes in the position of the mandible [18]. However, no effect on mandibular anterior displacement has also been reported [19].

Wider and longer dental arches are known outcomes of CHG treatment, and especially in the molar region, expansion is shown to be stable [17]. Earlier findings have shown that CHG treatment reduces the need of extractions and fixed appliance therapy [16]. Spaced dental arches after CHG treatment allow a more vertical canine eruption pattern in the maxilla compared with crowded dental arches [20, 21].

The appropriate timing of Class II treatment remains controversial. The early timing of orthodontic treatment reduces the risk of incisal trauma in children with Class II malocclusion [22, 23]. Early-timed CHG treatment provides larger and wider dental arches compared with later-timed CHG treatment in short-term follow-up, especially in males [15]. Early timing also reduces the need for later orthodontic treatment [24]. However, it often leads to two-phase treatment and longer mean total treatment time, which increases the total number of appointments [16]. There is no knowledge of the possible differences between early- and later-timed CHG treatment in dental arch changes based on long-term follow-up of randomized studies.

The aim of this study was to analyse the long-term influences of the timing of CHG treatment on the development of dental arches in a randomized setting. The hypothesis was that timing of the CHG treatment affects dental arch dimensions and stability of the outcome in long-term follow-up.

## Materials and methods

### Trial design

This study was a long-term follow-up of a previous randomized controlled trial. The trial design was a prospective, parallel-group, randomized (1:1 ratio) controlled trial. The trial was approved by the Regional Ethics Committee of Northern Ostrobothnia Hospital District (EETTMK: 46/2003) and health care centre supervisors. The trial is registered at ClinicalTrials.gov (NCT02010346). There were no changes in the methods after trial commencement. No harms were observed during this study. The protocol was not published before the screening. Consolidated Standards of Reporting Trials (CONSORT) 2010 guidelines were followed [25].

### Participants

Three public health care clinics from Northern Finland took part in this study. Two hundred and seventy 7-year-old children from birth cohorts were screened for the trial. The children were examined by general dentists and their eligibility for this study was considered by orthodontists in their local health care centres.

The inclusion criteria for this study were Angle Class II molar relationships, overjet over 6 mm, and deep bite. Exclusion criteria were inborn facial syndrome, facial asymmetry, the angle between palatal and mandibular line (PL-ML) over 35 degrees, earlier orthodontic treatment, obstructive sleep apnoea, and continuous airway infections.

**Figure 1.**
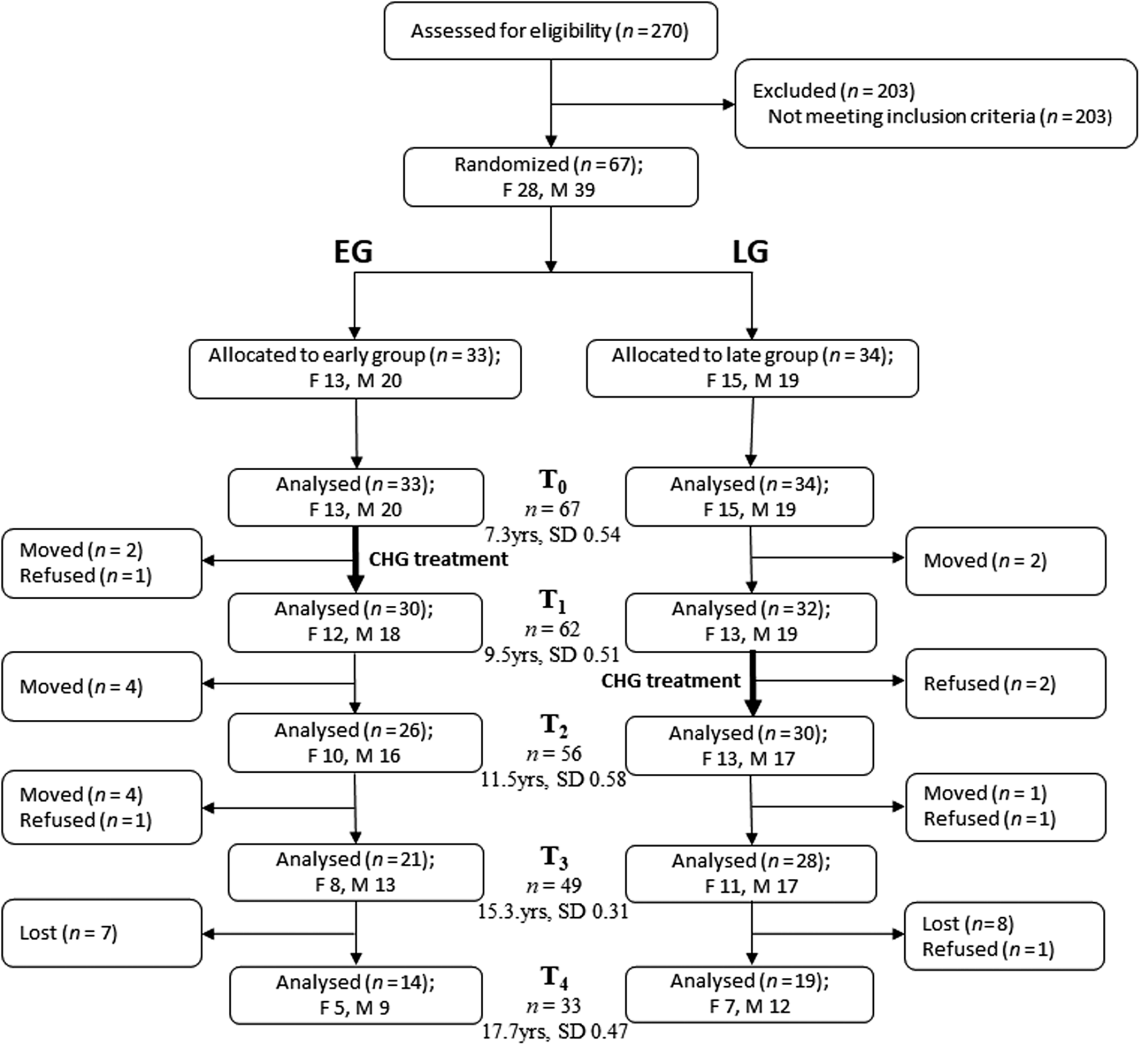
Flow chart of the participants in the trial (F: female; M: male). Dental casts at *T*_3_ or *T*_4_ were available for 7 females and 12 males in EG and 10 females and 17 males in LG, who are included in this study..

Figure 1 presents the flow of the participants from screening to the end of the study. Sixty-seven children met the inclusion criteria and took part in this study. The children were randomly divided into early group (EG, *n* = 33) and later group (LG, *n* = 34). Detailed information of the randomization has been published before [15, 26, 27]. Patients were recruited to this study from February 2004 to June 2008. The follow-up ended in June 2018. At the end of the study, the total number of participants was 33 (EG, *n* = 14; LG, *n* = 19).

Blinding was not possible for clinicians, patients, or their parents. All the participants were coded (J.J.), so patients, groups, or gender could not be identified when examining the data.

### Sample size calculation

The sample size calculation was based on the means and standard deviations (SDs) of the ANB angle values from a CHG trial with a similar study design [13]. GPower 3.1 Software with 80% power and 5% significance level was used in the calculation, resulting in a minimum of 11 participants in each group to reach sufficient power. The number of participants was increased to tolerate interruptions during the long-term follow-up.

### Treatment

The CHG therapy was started in the EG after the eruption of the first permanent upper molars. The treatment was carried out between time points *T*_0_ and *T*_1_. Among all participants, the CHG therapy was planned to be active until Angle Class I molar relationships were achieved (mean 1.6 years, SD 0.76), and reduced use was applied when necessary. The treatment protocol was accurately defined before the initiation of the study. The practitioners were calibrated before starting the study and regularly evaluated during the active treatment. All the patients were treated according to a similar treatment protocol until *T*_2_.

In the LG, the CHG treatment started 18 months later than in the EG at *T*_1_. The treatment protocol for the LG was identical to the treatment of the EG. The active phase lasted 1.4 years in the LG (SD 0.80). During the CHG treatment of the LG at *T*_1_–*T*_2_, there was no active orthodontic treatment in the EG.

The intraoral part of the facebow was expanded 5 mm. The long extraoral bow was bent 10 degrees upwards in relation to the intraoral bow. Molar bands with gingival tubes were used. The extraoral force magnitude was 500 g. Patients were instructed to use CHG for 8–10 h/night.

The need for further orthodontic treatment was considered individually for each patient after the active treatment phase at *T*_2_. The percentages of the additional orthodontic treatment were calculated of those 46 children of whom dental casts were available, and who participated either at *T*_3_ or at *T*_4_. Fixed appliance therapy in the upper dental arch was carried out among 53% of the participants in the EG and among 59% in the LG. Fixed appliance therapy in the lower dental arch was carried out among 37% in the EG and among 44% in the LG. Eruption guide appliance therapy was carried out among 47% in the EG and among 44% in the LG. Functional appliance therapy was carried out among 58% in the EG and among 41% in the LG. Extraction of some maxillary deciduous tooth was undertaken among 32% in the EG and among 22% in the LG. Extraction of some mandibular deciduous tooth was undertaken among 5% in the EG and among 19% in the LG. Extraction of deciduous tooth was not undertaken as a result of dental crowding. Extraction of maxillary permanent tooth was undertaken among 7% in the LG. Extraction of mandibular permanent tooth was undertaken among 11% in the LG. There were none of extraction of the permanent tooth in the EG.

### Drop-out analysis

Of the 67 participants, of whom dental casts were available, 21 dropped out before *T*_3_, and 46 participated either at *T*_3_ or at *T*_4_. There were no statistically significant differences at baseline between those who dropped out and those who stayed in the study according to group, gender, or dental arch measurements. Among females, the drop-out rates were 46% in EG and 33% in LG. Among males, the drop-out rates were 38% in EG and 11% in LG (*P* = .074).

### Study cast analysis

Alginate impressions and wax bite indexes in maximum intercuspal position were taken at time points *T*_0_ (7.3 years, SD 0.56), *T*_1_ (9.5 years, SD 0.50), *T*_2_ (11.5 years, SD 0.62), *T*_3_ (15.3 years, SD 0.30), and *T*_4_ (17.7 years, SD 0.66). Dental casts were scanned into 3D models (3Shape, R700^TM^ Orthodontic Scanner, Denmark) and measured with an analyser programme (3Shape, Ortho Analyzer^TM^ 2012, Denmark).

**Figure 2.**
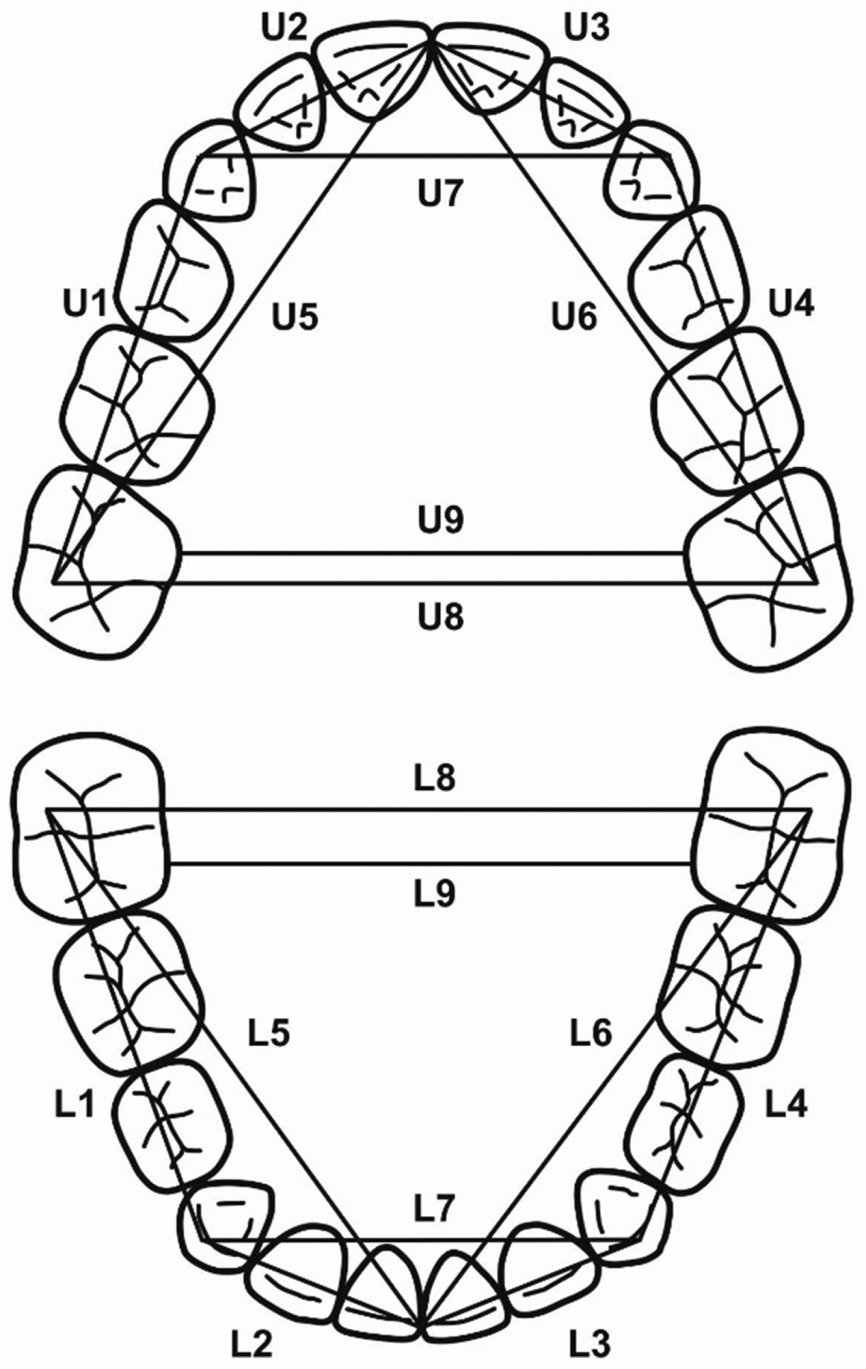
Landmarks for the dental cast analysis. U1, U4, L1, L4: the distance between the distobuccal cusp of the first permanent molar and the highest point of the canine in the same quadrant. U2, U3, L2, L3: the distance between the highest point of the canine and the most mesial point of the central incisor in the same quadrant. U5, U6, L5, L6: the distance between the distobuccal cusp of the first permanent molar and the most mesial point of the central incisor in the same quadrant. U7, L7: the distance between the highest points of canines. U8, L8: the distance between the distobuccal cusps of the first permanent molars. U9, L9: the shortest distance between the palatal or lingual surfaces of the first permanent molars.

The linear measurements are shown in Figure 2. The landmarks for measurements were the highest points of distobuccal cusps in the first molars, tips of canines, and the most mesial points of the central incisors. A more detailed description of the measurements is presented in our previous study [15].

All scanning and measuring were performed by one of the authors (M.H.). Twenty dental casts were measured twice at 2-week intervals to calculate the error of the method using interclass correlation coefficient (ICC).

### Statistics

Mean values (mm) and SD for all dental arch measurements at different time points (*T*_0_–*T*_4_) were reported according to group and gender. General linear models (GLMs) for repeated measures were conducted using all five time points and using gender and group as between-subject factors, a separate model for each measurement. Changes from time point *T*_0_, and from previous time points were calculated and are presented as figures. *P*-values <.05 were considered as statistically significant. All reported *P*-values were from 10 different models, and no mathematical correction for multiple comparisons was made. Statistical analyses were performed using IBM SPSS version 27 (SPSS, Inc., Chicago, IL, USA).

### Method error analysis

The ICC values ranged from 0.971 to 0.999 in the repeated measurements of dental casts. The level of intra-rater reliability was excellent.

## Results

### Changes in maxillary dental arch

Based on GLM for repeated measures (five time points), there were several significant differences between EG and LG at *T*_0_–*T*_4_ (Tables 1 and 2). The changes in the upper dental arch were not gender-related during the follow-up.

**Table 1.** Mean values (mm) and SD for dental arch measurements at time points *T*_0_–*T*_4_ according to group and gender.

		*T*_0_		*T*_1_		*T*_2_		*T*_3_		*T*_4_	
		EG	LG	EG	LG	EG	LG	EG	LG	EG	LG
Females											
U1+U2+U3+U4	Mean	86.7	87.2	93.8	88.4	92.8	91.5	92.7	88.6	92.3	88.6
	SD	3.5	1.9	5.2	1.9	5.5	5.4	4.7	5.5	3.6	6.3
U5+U6	Mean	80.9	81.3	88.2	82.7	87.8	85.0	86.3	82.9	86.1	82.7
	SD	2.9	2.4	5.0	4.0	4.9	5.2	3.5	5.2	2.8	5.6
U7	Mean	31.2	31.7	34.5	32.3	33.7	33.1	34.6	34.0	34.9	34.5
	SD	1.7	2.3	2.3	1.6	3.1	1.7	1.9	1.4	0.8	1.3
U8	Mean	49.4	49.6	52.4	50.1	52.4	51.5	52.8	51.5	52.8	51.8
	SD	1.5	2.8	2.9	2.9	3.1	2.6	2.4	2.8	1.9	2.8
U9	Mean	31.0	31.9	33.9	31.8	33.8	33.1	34.4	33.3	34.3	33.4
	SD	0.9	2.8	2.4	2.8	2.3	2.8	2.5	2.8	1.5	2.6
L1+L2+L3+L4	Mean	80.4	80.3	82.3	81.0	82.0	78.0	79.1	75.6	78.5	76.2
	SD	3.4	2.6	4.5	2.7	4.4	5.6	4.3	4.4	2.2	5.1
L5+L6	Mean	75.0	75.0	76.3	75.2	75.5	73.4	73.8	71.0	73.3	71.8
	SD	3.4	2.6	4.1	3.6	3.5	4.7	3.3	4.1	1.8	4.8
L7	Mean	23.5	24.0	25.3	24.9	25.9	25.0	24.8	24.2	25.0	25.0
	SD	1.4	1.8	1.8	1.9	1.4	1.5	1.7	1.0	1.2	0.8
L8	Mean	45.6	44.6	46.8	44.9	47.1	45.3	47.2	45.2	46.9	46.0
	SD	2.3	2.4	2.3	2.7	2.4	2.7	1.8	2.4	1.8	2.8
L9	Mean	31.0	30.8	31.6	30.8	31.8	30.5	31.7	30.2	31.3	31.2
	SD	1.5	2.4	1.4	2.8	1.7	2.9	1.2	2.9	1.7	3.5
Males											
U1+U2+U3+U4	Mean	88.0	90.2	97.3	92.5	95.8	95.5	94.3	93.5	93.7	91.9
	SD	9.0	4.2	4.0	3.8	3.9	6.1	3.8	3.6	4.0	3.7
U5+U6	Mean	82.7	85.2	91.0	87.1	89.2	88.9	88.3	87.1	86.6	85.4
	SD	8.6	4.7	4.0	5.0	3.6	5.7	3.3	4.0	2.9	3.9
U7	Mean	32.3	31.7	36.1	33.1	34.1	35.3	35.8	36.0	36.0	36.0
	SD	3.1	2.0	2.9	1.5	2.3	2.2	2.0	1.6	2.5	1.9
U8	Mean	51.3	51.3	54.0	52.3	54.2	52.7	54.4	53.9	54.1	54.2
	SD	3.0	2.8	1.9	3.2	1.9	2.9	1.6	3.1	1.5	3.1
U9	Mean	32.8	33.0	34.8	33.3	34.9	33.7	35.1	35.1	34.7	35.7
	SD	2.8	3.3	2.1	3.6	1.7	2.7	1.5	3.0	0.9	3.2
L1+L2+L3+L4	Mean	81.0	82.2	84.7	82.2	83.5	81.5	81.7	80.4	80.8	79.9
	SD	8.0	4.1	2.1	4.5	2.8	4.8	3.1	3.6	3.7	4.0
L5+L6	Mean	75.4	76.7	78.4	76.8	78.6	76.7	76.8	75.1	74.8	74.7
	SD	7.5	4.2	2.3	5.1	2.3	4.9	2.7	3.4	3.3	3.6
L7	Mean	23.8	23.8	25.0	25.1	26.0	26.6	25.5	26.1	25.5	26.1
	SD	2.6	2.0	2.8	2.7	2.1	2.6	2.0	1.7	1.8	2.1
L8	Mean	47.0	46.7	48.6	46.7	49.6	47.1	49.1	47.9	48.7	48.7
	SD	2.5	2.3	1.7	2.4	2.0	2.7	2.1	2.9	1.9	3.4
L9	Mean	32.0	31.5	33.0	31.5	33.9	32.0	33.1	32.8	32.7	33.2
	SD	2.3	2.1	2.0	2.3	1.9	2.6	1.9	2.6	1.8	3.2

*P*-values have been stated in Table 2.

**Table 2.** *P*-values for the significance of change, group difference in change (Change × Group), gender difference in change (Change × Gender), and independent effects of group and gender based on GLM for repeated measures (five time points) on different dependent variables.

Dependent	Change	Change × Group	Change × Gender	Group	Gender
U1+U2+U3+U4	<.001	.048	.839	.924	.166
U5+U6	<.001	.035	.966	.386	.112
U7	<.001	.001	.112	.700	.108
U8	<.001	.002	.776	.506	.080
U9	<.001	.002	.897	.840	.131
L1+L2+L3+L4	<.001	.560	.031	.206	.134
L5+L6	<.001	.105	.020	.248	.123
L7	<.001	.986	.072	.821	.874
L8	.004	.290	.925	.123	.030
L9	.151	.223	.513	.234	.065

**Figure 3.**
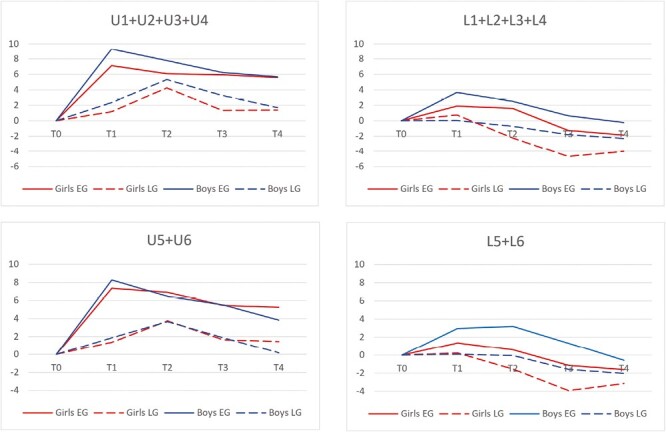
Changes in total dental arch lengths (mm) between time points *T*_0_–*T*_4_ according to group and gender. At *T*_0_, mean age of the participants was 7.3 years. The gap between time points was on average 2.2, 2.0, 3.8, and 2.4 years. Values are based on 46 participants who attended either *T*_3_ or *T*_4_. *P*-values are given in Table 2.

The total upper dental arch length (U1+U2+U3+U4) increased more in EG compared with LG at *T*_0_–*T*_4_ (*P* = .048; Fig. 3). The change at *T*_0_–*T*_4_ was larger for EG compared with LG in both genders. The total change was greater in EG immediately after the CHG treatment (*T*_1_) compared with LG (*T*_2_). The change was more stable in EG at the end of follow-up (*T*_4_), while there was a significant decrease in LG.

The upper bilateral molar-incisal distance (U5+U6) showed more increase in EG compared with LG at *T*_0_–*T*_4_ (*P* = .035; Fig. 3). The change at *T*_0_–*T*_4_ was greater in EG compared with LG in both genders. The total change was larger in EG immediately after the CHG treatment (*T*_1_) compared with LG (*T*_2_). The change was more stable in EG at the end of the trial (*T*_4_) while there was more decrease in the LG.

**Figure 4.**
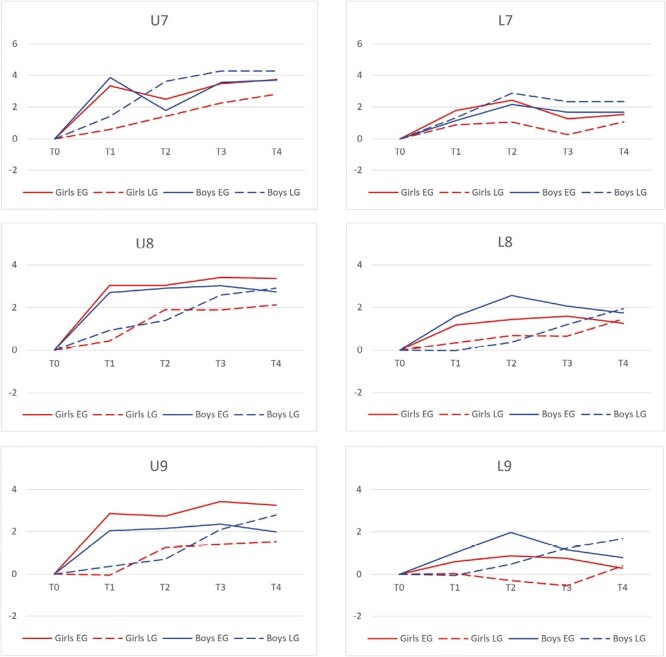
Changes in dental arch widths (mm) between time points *T*_0_–*T*_4_ according to group and gender. At *T*_0_, mean age of the participants was 7.3 years. The gap between time points was on average 2.2, 2.0, 3.8, and 2.4 years. Values are based on 46 participants who attended either *T*_3_ or *T*_4_. *P*-values are given in Table 2.

The width between the upper canines (U7) increased more in EG compared with LG during the follow-up (*P* = .001; Fig. 4). The transversal dimension between canines decreased in EG and increased in LG at *T*_1_–*T*_2_. The intercanine width showed the most increase in LG males and the least increase in LG females at *T*_0_–*T*_4_.

The transversal dimensions between the first upper molars (U8, U9) increased in both groups, but the EG reached the final width at *T*_1_, while the LG reached the width at *T*_4_. The total increase was relatively similar in both groups in *T*_0_–*T*_4_ (*P* = .002, *P* = .002; Fig. 4). The widths (U8, U9) increased the most among EG females and the least among LG females at *T*_0_–*T*_4_. The width between upper first molars was relatively stable in both genders in EG at *T*_1_–*T*_4_ and in LG females at *T*_2_–*T*_4_ after CHG treatment but increased significantly in LG males during entire the follow-up at *T*_0_–*T*_4_.

### Changes in mandibular dental arch

The mandibular dental arch length showed significant long-term differences between males and females at *T*_0_–*T*_4_ (Tables 1 and 2). The long-term changes in the mandibular dental arch were not group-related during the follow-up.

The total lower dental arch length (L1+L2+L3+L4) showed more decrease in females compared with males at *T*_0_–*T*_4_ (*P* = .031; Fig. 3). The lower dental arch was longer in males compared with females after CHG treatment. The long-term changes in mandibular dental arch were not group related (Tables 1 and 2).

The lower bilateral molar-incisal distance (L5+L6) decreased more in females compared with males at *T*_0_–*T*_4_ (*P* = .020; Fig. 3). The lower dental arch showed more length in males compared with females after CHG treatment. The long-term changes in lower dental arch were not group-related (Tables 1 and 2).

No statistically significant long-term differences between groups or genders were found in the transversal lower dental arch dimensions (Tables 1 and 2).

## Discussion

### Main findings

This long-term study showed that the timing of CHG treatment has effects on dental arch changes, and both genders benefit from an earlier approach. Maxillary dental arches were longer after CHG treatment in EG compared with LG during the long-term follow-up period. The final width of the maxillary dental arch was also reached earlier in EG than in LG.

### Interpretations

An interesting finding was that the total maxillary dental arch length was achieved earlier, the length extension persisted better in the long term, and more space was gained in EG compared with LG (Fig. 3). Both dental arch length measurements (U1+U2+U3+U4 and U5+U6) showed a significant increase in EG during the follow-up. The increase in the total arch length in EG between *T*_0_ and *T*_1_ is in accordance with previous studies [13–16]. This study, however, showed that in EG, the gained length was more stable in the long term after the active phase of treatment than in LG. A previous study on normal occlusion has shown that the total upper dental arch length increases between 7 and 10 years of age and decreases almost to its former extent from 10 to 16 years of age [2]. The decrease in maxillary dental arch length during the late mixed dentition is partially a consequence of the first molar drift mesially during the replacement of primary molars. The found decrease in LG after the active phase of treatment corresponds to the development of normal dental arch [2], and the gained space was substantially less than that obtained in the early treatment group.

The eruption stage of the maxillary second molars reduces distal movement of the maxillary first molars [28, 29]. In EG, the maxillary second molar crowns are mineralizing at the time of active treatment, while in LG, the root development of the maxillary second molars is in progress during the active phase of treatment [30]. This could be a possible explanation for the more effective arch length increase in EG than in LG, as the pressure of the developing maxillary second molars is smaller. The gained space was more stable in the EG, which could be explained by easier distal movement of developing second molar buds as a consequence of CHG treatment. A previous study has shown that unerupted maxillary second molars move distally alongside the first molar movement during CHG treatment, although the developmental stage of the second molars was not found to be related to distal movement of the first molars [31]. We suppose that in LG, the arch length increase is restricted due to more advanced developmental stage of the maxillary second molars, and the gained space relapses due to the pressure from erupting second molars.

This study showed that the intermolar width in the maxillary dental arch was stable, and the outcome was reached earlier in EG compared with LG. Studies on normal occlusion have demonstrated the long-term stability of dental arch widths after adolescence [1, 2]. A decrease in dental arch intermolar widths has been shown to be less than 1 mm between 15 and 32 years of age [1]. Thus, dental arch widths are considered to be stable in the long term. The CHG has been stated to provide wider dental arches compared to controls without CHG treatment in long-term follow-up [16]. CHG widens dental arches by an expanded inner bow and by reducing buccal soft tissue pressure. The present results did not show clinically significant differences in intermolar widths between EG and LG at the end of the follow-up. However, EG reached the maxillary intermolar widths earlier, thus creating space in the maxillary dental arch before late mixed dentition.

Dental crowding and reduced intercanine width in the maxilla are associated with buccally displaced canines [32]. In CHG use, the timing of treatment is important because early use creates additional space before late mixed dentition and contributes to the alignment of erupting canines and premolars. CHG treatment has been shown to make the eruption pattern of maxillary canines more vertical [20, 21]. The present results showed that maxillary intercanine width was reached earlier in EG compared with LG, but at the end of the follow-up, there were no significant differences between the groups. There was a decrease in intercanine width in EG after the active phase of CHG treatment. This decrease could be due to different eruptional stage of permanent canines between the groups.

The present results showed that long-term changes in mandibular dental arch lengths were gender-related, not group-related. In EG, mandibular dental arch length increased spontaneously during the active phase of treatment, especially in males. Instead, in LG females, mandibular dental arch length started to decrease during the active phase of treatment. The present results showed that EG males benefited the most and LG females the least from the CHG treatment in terms of mandibular arch length increases. A previous study found wider mandibular arches and less decrease in the total mandibular arch length in the long term among patients treated with CHG compared with patients without CHG treatment [16]. Similar short-term effects of CHG treatment on mandibular dental arch have been reported [13, 15, 17]. Although early timing of CHG treatment provides wider mandibular dental arches in the short term compared with later timing [15], the current results indicate that timing has no significant effect on lower dental arch widths in the long term. However, a favourable short-term effect may be clinically significant during late mixed dentition, especially in crowded mandibular arches.

CHG treatment is usually two-phase treatment aiming to reduce the complexity of later orthodontic treatment. The majority of the participants in both groups needed second-phase treatment. Fixed and eruption guidance appliances may affect dental arch dimensions, but the prevalence of these appliances was similar in both groups. The rigid functional appliance generally has no effect on dental arch dimensions. Increased need for extractions in LG compared with EG confirms that the earlier approach decreases the need for permanent extractions. The effect of extractions on dental arch length was statistically tested by excluding participants with permanent tooth extractions. Because no significant influence on the results was found, dental casts with permanent tooth extractions were not excluded from the final statistical analysis.

In this study, the force of 500 g for the use of CHG was chosen for both groups. According to the latest studies, the use of light force (300 g) is recommended in CHG treatment [17, 31, 33]. Light force improved the adherence to CHG instructions compared with the heavy force of 500 g [33]. The use of light force also induced slightly wider intermolar dimensions in both dental arches compared with the heavy force [17]. Thus, the use of lighter force may improve the outcome along with better adherence to CHG instruction, ensure compliance with later orthodontic treatment, and prevent patients from dropping out.

When evaluating the long-term effects of CHG treatment, it is important to be conscious of normal dental arch development. Longitudinal studies of normal occlusion have shown a tendency for length decrease and maxillary width stability during follow-up. It has been stated that normal dental arch development is difficult to distinguish from relapse after orthodontic treatment [34]. The present results in two different age groups clearly showed that the gained length persisted better in earlier-treated subjects than those treated later. The benefits of the earlier timing of CHG treatment compared with later timing have clinical relevance. Thus, it is suggested that CHG treatment with early timing is a viable method in cases where Angle Class II malocclusion is combined with dental crowding.

### Limitations and generalization

The weakness of this trial is that for ethical reasons, an untreated control group cannot be used.

The sample size is based on power analysis, but a greater sample size would provide a higher level of evidence. The analyses are based on values of 46 participants who attended either *T*_3_ or *T*_4_ and of whom dental casts were available, to minimise the effects of the drop-outs to the results. In a long follow-up trial such as the present one, drop-outs are unavoidable, but they may also cause bias in the results. Several drop-outs affect negatively the power, which may cause bias. Especially, the gender subgroups are relatively small, and the effect of gender must be interpreted with caution.

CHG is a removable appliance, and co-operation is essential for successful treatment. Orthodontists cannot monitor how patients follow instructions. Two of the participants in both groups did not reach Class I relation of the molars during CHG therapy.

The main strength of this trial was the long follow-up period. There are no previous studies evaluating the long-term effects of CHG on dental arches. Thus, the present results provide valuable information in the field of long-term treatment effects.

The present results confirm that CHG is a viable appliance in the treatment of CII malocclusion between 7.5 and 11.5 years of age.

## Conclusion

The timing of CHG treatment influences dental arch changes in the long term. Both genders benefit from earlier CHG treatment, males even more than females, especially in the short term. The maxillary dental arches remained longer, and the final width was gained earlier in EG compared with LG. Although mandibular dental arch length and width increased during late mixed dentition in EG compared with LG, the long-term length changes in mandible dental arch were gender related. The results showed that in terms of total mandibular arch length increases, EG males seem to benefit more and LG females less from CHG treatment.

Thus, these findings support our hypothesis that CHG timing affects dental arch dimensions and the stability of the outcome in long-term follow-up. However, if early treatment is considered, there seem to be some benefits in starting the treatment right after the eruption of the first maxillary molars in CII malocclusion.

## Data Availability

The data underlying this article will be shared on reasonable request to the corresponding author.
